# Alcohol Use and Church Attendance Among Seventh Through Twelfth Grade Students, Dominican Republic, 2011

**DOI:** 10.1007/s10943-012-9663-0

**Published:** 2012-11-21

**Authors:** Michael N. Dohn, Santa Altagracia Jiménez Méndez, Maximinia Nolasco Pozo, Elizabet Altagracia Cabrera, Anita L. Dohn

**Affiliations:** 1 Programa de Salud Comunitaria, Clínica Esperanza y Caridad, Iglesia Episcopal Dominicana, Apartado 509, C/ Sánchez #9, esq. Freddy Gaton Arce, Sector Miramar, San Pedro de Macorís, 21000 Dominican Republic; 2 Society of Anglican Missionaries and Senders, PO Box 399, Ambridge, PA 15003 USA

**Keywords:** Religion, Adolescent health, Alcohol abuse, Ethanol, Health promotion, Primary prevention, Social networks, Peer education, Church attendance, High school

## Abstract

Excessive alcohol consumption increases the years of life lost to premature death and disability worldwide. Religion is a mitigating factor in alcohol consumption. A survey in the Dominican Republic showed increasing church attendance by middle and high school students (*N* = 3,478) was associated with a delay in age at first alcoholic drink, fewer students who had consumed alcohol in the past month (current drinkers), lower alcohol consumption levels, fewer episodes of inebriation, and less heavy episodic alcohol consumption (all *P* < 0.0001). The results suggested that it may be useful to conceive of church-attending youth as a subset of the adolescent social network when planning primary alcohol prevention programs for young people.

## Introduction

The excessive consumption of alcohol and episodic heavy drinking cause increases in the years of life lost to premature death and disability (DALY’s or disability adjusted life years) worldwide, and these effects are worsening (Jernigan 2002; Monteiro 2007). Alcohol accounts for the largest number of lost DALY’s in the Americas (Monteiro 2007; Rehm and Monteiro 2005). Alcohol is the leading risk factor for lost DALY’s in all 34 countries of the Americas except Bolivia, Ecuador, Guatemala, Haiti, Nicaragua, and Peru in which alcohol runs second to smoking (Rehm and Monteiro 2005).

Alcohol affects health in various ways: direct pathophysiological effects, lessening of agility and coordination, changes in moods and emotions, and mitigation of mental and reasoning abilities (Jernigan 2002; Monteiro 2007; Rehm and Monteiro 2005). Thirty-nine codes of the International Classification of Diseases are entirely attributable to alcohol (Rehm and Monteiro 2005). Alcohol is a contributing cause for a number of additional chronic and infectious conditions as well as many categories of intentional and unintentional injuries (Jernigan 2002; Rehm and Monteiro 2005).

The most effective measures to decrease alcohol-related health consequences impact alcohol consumption at the population level; these measures include taxation, control of alcohol sales (including minimum-age limits), and drinking-driving prohibitions (Brand et al. 2007; Jernigan 2002; Monteiro 2007). However, “a policy focus on alcohol problems and the resources to alleviate them remain scarce in the developing world” (Jernigan 2002, p. 23). Stakeholders (from local vendors to transnational corporations) and international trade agreements may not place public health interests as the highest priority and can complicate effective policy implementation (Jernigan 2002; Monteiro 2007).

Individual behavior change models, particularly primary prevention strategies with young people, have generally not shown good long-term results (Foxcraft et al. 2008; Jernigan 2002). However, a few strategies have shown promise (Marlatt and Witkiewitz 2002). Harm reduction strategies are at least as effective as abstinence programs, and all interventions could be improved with better definition of the relevant factors related to adolescent development and behavior (Foxcraft et al. 2008; Marlatt and Witkiewitz 2002; Spoth et al. 2008).

Religion is a common element of civic life in the Dominican Republic. A centrally placed representation of the Bible is constitutionally mandated on the Dominican national coat-of-arms (Gobierno de la República Dominicana 2002). The influence of Christianity is apparent in civic life in the names of many small businesses and in the use of Biblical quotes on bulletin boards in public schools and in government-sponsored national safe driving campaigns. The level of religious affiliation is high, 95 % Roman Catholic being a common estimate (CIA 2011). However, the degree to which this represents personal beliefs or an administrative assumption is uncertain. For example, the Dominican national electronic database for people being treated for HIV/AIDS uses “Roman Catholic” as the default value for all patients. On an individual level, during clinical reviews of systems, people often respond to questions about alcohol use with a variation of “I don’t drink. I’m a Christian,” linking religious beliefs to a behavior with health implications.

The extent to which participation in religious services relates to age at first drink, quantity of alcohol consumed, episodes of inebriation, episodic heavy drinking, and alcohol-related problems among adolescents in the Dominican Republic is uncertain. Religion could be a relevant factor in planning primary prevention activities for youth in Latin America and elsewhere. This study examines some elements of alcohol use in the Dominican Republic and self-reported church attendance of youth in grades 7 through 12.

## Materials and Methods

The survey instrument was based on the alcohol core module of the Spanish-language version of the Global School-Based Student Health Survey (WHO 2009a). Modifications included language changes to reflect common local usage and addition of several original items and items from other sources (WHO 2009b). Other than age, all items were multiple choice. The questionnaire was limited to 16 items to minimize respondent burden and fatigue (Smith et al. 2005a).

The questionnaire collected data on basic demographics (age, gender, grade level); indication of spiritual life (church attendance of students and their families); the family environment related to alcohol (alcohol in the home, if household members drink and how often); age at alcohol initiation (i.e., first drink); student alcohol consumption (time of last drink, frequency and quantity of recent alcohol consumption); students’ sources of alcohol; and the number of episodes of inebriation, of episodic heavy drinking occurrences, and of alcohol-related family, social, and school problems.

“Standard drink” definitions vary internationally (Devos-Comby and Lange 2008). This study used the definitions from the original survey source (WHO 2009a). These “standard drinks” had different alcohol contents and vary from the “standard drink” definitions used by the Centers for Disease Control and Prevention that are defined by equal alcohol content (CDC 2012), for example. This variance may have had an effect on the students’ responses, though it is uncertain whether the students were aware of the alcohol content of the drinks.

The questionnaire was evaluated for face, content, and construct validity (Browne 2005a). A standard to check criterion validity was not available. Face validity was evaluated through review by the authors and professional educators. Content validity was evaluated by piloting the questionnaire with 122 students from seventh through twelfth grade, and two follow-up focus groups in which each item was reviewed. Construct validity was evaluated by examining patterns for known groups (more alcohol consumption reported with increasing grade level, for example), as well as convergence and discriminatory characteristics. Test–retest reproducibility was evaluated through the focus groups in which students indicated that they would maintain their original responses.

One item, evidently designed to measure problems associated with a student’s personal alcohol consumption (WHO 2009c, d), captured alcohol-related family and social problems in the lives of students who did not drink; that item was left unchanged in the final questionnaire.

The questionnaire was administered to a convenience sample of students from seventh through twelfth grades in nine schools: three public and six private (three secular and three church-related) from 17 February through April 14, 2011. Administration was class-by-class using an informed consent process approved by the human subjects review committee of Clínica Esperanza y Caridad. Questionnaires consisted of three sheets of paper printed on both sides with introductory information on page one and questions on pages two through six (see "Appendix"). All questionnaires were administered by one of five trained personnel who were all first-language Spanish speakers. Questionnaires were distributed to all students in each class who placed them into individual envelopes when they finished. Students deposited the envelopes into a collection bag for each class.

During data entry, grade level and school were systematically entered for each student. Multiple responses by a student to an item were coded as missing answers.

Statistics were both descriptive and analytical. Continuous data were evaluated by *t* test for two groups and one-way analysis of variance for multiple groups. Paired ordinal data were evaluated with the Wilcoxon signed rank test. Ordinal data were compared for two groups by Wilcoxon rank sum test and for multiple groups by the Kruskal–Wallis test. Overall Type I error was controlled to a level of 0.05 for multiple pair wise comparisons using Scheffe procedures. Kaplan-Meier survival analysis and proportional hazards regression were used for censored data (such as age at first alcoholic drink). A commercial statistics program (Stata/IC 10, StataCorp, College Station, Texas, USA) was used for calculations with some 95 % confidence intervals (CI) calculated by hand (Gardner and Altman 1989). *P* values less than 0.05 were considered statistically significant.

## Results

The survey was administered to 3,480 students. Two surveys were excluded from the analysis as not interpretable, leaving a sample size of 3,478 surveys. The final sample included three public schools (2,091 students), three secular private schools (455 students), and three church-related schools (932 students). To obtain this sample, eleven schools were approached; the administrations of two secular private schools declined participation and replacement schools were surveyed. All schools were within District 05-02 (San Pedro de Macorís Oeste). The results reported for current drinkers, average daily consumption, binge drinking, episodes of inebriation, and alcohol-related problems have been directly standardized to the District 05-02 enrollment figures for grades 7–12 for the 2009–2010 school year based on school category (Ministerio de Educación 2011).

Eleven students (0.03 %) chose not to respond to the survey (ten students did not respond to any items, and one student answered only the demographic portion); their surveys were retained in the analysis as missing answers. Overall, 2,981 students (85.7 %) responded to at least 13 questions and 2,351 students (67.6 %) responded to all questions. Mean response rate for items was 93.3 % (range 88.9–96.8 %). The median number of items answered was 15 for all school types and for male and female students.

Gender was indicated by 3,368 students (96.8 %) with 46.3 % male and 53.7 % female students. Age was provided by 3,337 students (95.9 %) and was different by each grade level (*P* < 0.0001), with the exception of a group of three accelerated classes (combining two grade levels) that had a mean age the same as the eleventh grade; students in the accelerated classes were included as eleventh graders in the analysis. Reported ages in the sample were used to calculate age at first alcoholic drink and percentage of current underage drinkers; otherwise grade level was utilized as a surrogate for age.

In terms of church attendance, the students attend less frequently than their families (*P* < 0.0001) (Table 1). Responses indicated that alcohol is never stored in the homes of 1,680 students (52.4 %), sometimes in 1,231 (38.4 %), and always in 295 (9.2 %). One or more members of the household were reported to drink alcohol by 2,269 students (71.8 %). Frequency of alcohol consumption by household members was Never 796 (25.3 %); less than once a month 719 (22.9 %); several times a month 832 (26.5 %); 1–2 days per week 620 (19.7 %); 3–5 days per week 98 (3.1 %); and daily 80 (2.5 %).

**Table 1:** Comparison of frequency of church attendance between students and their families (*N* = 3,345)

Frequency of family church attendance (*n*)	Frequency of student church attendance (*n*)					
	Never	Once in awhile	Monthly	Weekly	More than once a week	Total, *n* (%)
Never	16	59	3	6	13	97 (2.9)
Once in awhile	23	520	16	70	95	724 (21.6)
Monthly	4	73	29	10	18	134 (4.0)
Weekly	10	289	31	181	88	599 (17.9)
More than once a week	25	853	61	188	664	1791 (53.5)
Total, *n* (%)	78 (2.3)	1,794 (53.6)	140 (4.2)	455 (13.6)	878 (26.2)	

Several items were associated in predictable ways. While this supports the internal validity of the questionnaire, it also suggests possible confounding of those factors. Attending a church-related school was related to the church attendance patterns for both students and their families (both *P* < 0.0001). Similarly, the students’ and families’ church attendance patterns also related to alcohol storage in the home, household alcohol consumers, and frequency of alcohol consumption by household members (all *P* < 0.0001); pair wise comparisons indicated that these associations were due to the effect of those attending church more than once a week. Female students tended to attend church more frequently than male students (*P* = 0.059).

**Fig. 1:**
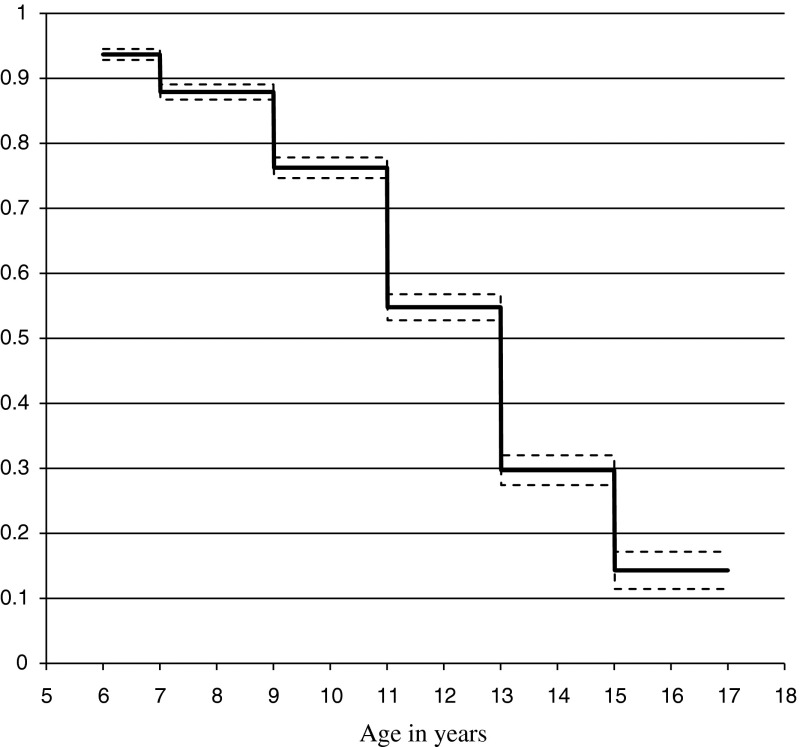
Estimated proportions of young people by age groups who have not had an initial alcoholic beverage (*dotted lines* show 95 % confidence limits)

Students were most likely to have their first alcoholic drink between ages 11 and 15 years (Fig. 1). Age at first alcoholic drink was delayed related to female gender (relative risk 0.84, CI 0.78 to 0.91) and more frequent church attendance by the student (relative risk 0.88, CI 0.85 to 0.91, both *P* < 0.0001). Church attendance by the family was not associated with age at first alcoholic drink (*P* = 0.34).

About one-third (34.7 %) of the students were current drinkers (having consumed alcohol in the past month). Increasing church attendance by students was associated with decreased current drinking (*P* < 0.0001). The frequency of family church attendance was not associated with current student drinking (*P* = 0.42). Even with the delay in first alcoholic drink for female students, the proportion of current drinkers was not different by gender (male 34.7 %, female 34.5 %; *P* = 0.91). Current drinkers varied by grade level: seventh, 19.1 %; eighth, 24.0 %; ninth, 26.4 %; tenth, 39.0 %; eleventh, 39.1 %; and twelfth, 44.4 % (*P* < 0.0001). Within the sample population, 82.9 % of current drinkers were under 18 years of age.

The average daily consumption among the current drinkers on days that they did consume alcohol was less than one drink, 65.7 %; one drink, 12.7 %; two or three drinks, 11.5 %; and four or more drinks, 10.1 %. Female students reported consuming less than male students (*P* = 0.034). Increasing church attendance by students (*P* < 0.0001) and by their families (*P* = 0.004) was associated with lower quantities of alcohol consumption.

Students reported episodes of inebriation: None, 65.1 %; one or two episodes, 28.3 %, three to nine episodes, 4.0 %; and ten or more episodes, 2.6 %. Female students reported less inebriation than male students (*P* < 0.0001). Increasing church attendance by students (*P* < 0.0001) and by their families (*P* = 0.0095) was associated with less inebriation.

Students’ reports of heavy episodic “binge” drinking behavior (four or more drinks for female students and five or more for male students) indicated that 68.3 % had never binged; 20.2 % had one binge episode; 7.0 % had two to four episodes; and 4.6 % had five or more. Male and female students were equally likely to report two or more binge episodes (*P* = 0.69), students attending church more frequently were less likely to binge (*P* < 0.0001), and family church attendance was marginally associated with less binge behavior (*P* = 0.07).

The usual source of alcohol was identified by 1,340 students: 36 % obtain alcohol from their friends, 32.2 % buy it in a store or on the street; 14.4 % get it from their homes; 5.1 % make it themselves; 4.5 % have someone buy it for them; 1.3 % steal it; and 6.6 % get it another way. Of the 432 students who report buying their alcohol, 333 (78.5 %) gave their ages as under 18 years old, the legal age for alcohol purchase here.

Only a small proportion of students (7.9 %) reported having had problems with family or friends, having missed school or having gotten into fights because of alcohol. Multiple episodes (three or more) were reported by 1.9 % of the students. Of the students reporting alcohol-related problems, 9.3 % did not consume alcohol themselves.

## Discussion

There are multiple determinants of adolescent alcohol consumption, including health beliefs, self-identity formation, advertising and media exposure, public policy, and social networks (Anderson et al. 2009; Jernigan 2002; Masterman and Kelly 2003; Smith and Foxcraft 2009; Spoth et al. 2008).

Adolescent religiosity is protective against health risk behavior and alcohol misuse for youth in North America, South America, and Eastern Europe (Jernigan 2002; Piko and Fitzpatrick 2004; Sanchez et al. 2011; Stevens-Watkins and Rostosky 2010; Stritzke and Butt 2001; Zarzar et al. 2012). However, a review of the literature on adolescent religiosity, spirituality, health attitudes, and health behaviors found inconsistent theoretical bases and a lack of standard operational definitions of religiosity (Rew and Wong 2006). An attempt to separate religiosity into a public domain (frequency of attendance at religious services and participation in youth group activities) and a private domain (frequency of prayer and importance of religion) found that both domains were protective against youth risk behaviors (Nonnemaker et al. 2003). This report examining adolescent alcohol use and self-reported frequency of church attendance lies within the public religiosity domain, a domain that is perhaps more indicative of the young people’s social network than any particular religious beliefs.

Pertinent adolescent social networks affecting alcohol consumption include family and peers (Kristjansson et al. 2010; Kuntsche and Kuendig 2006). This study suggested that church participation represented a subset of the peer social network related to adolescent alcohol use.

Within the area of family networks, family-focused interventions for young people can result in delayed alcohol initiation and decreased alcohol consumption (Foxcraft and Tsertsvadze 2011); however, in general, family-based programs have not shown effects for young people 16 years and older (Spoth et al. 2008). A review of 145 critical incidents of adolescent alcohol drinking in the Netherlands suggests that parents have a minor influence on curtailing problematic drinking behavior, frequently being unaware of alcohol-related incidents (Van Hoff et al. 2011). In the present study, the students’ church attendance related to measures of alcohol use (including age at initial drink), while there were weaker or no associations with their families’ church attendance pattern. These findings suggest a waning influence of the family on older adolescent behavior.

Alcohol use by peers and friends, as well as gaining their respect or approval, is a factor in adolescent alcohol consumption (Kristjansson et al. 2010; Preston and Goodfellow 2006; Trucco et al. 2011). Young adolescents are particularly susceptible to exposure to a peer drinking network (Kelly et al. 2012). The perceived status or popularity of the influential peer or friend is also a factor (Bot et al. 2005); of interest is that presentation of anti-alcohol messages are more effective when coming from peers and friends who are seen as more popular (Teunissen et al. 2012). Paradoxically, popularity increases for adolescents who drink with their peers (Balsa et al. 2011).

Adolescent social networks influence initiation of alcohol consumption and drinking behavior (Ali and Dwyer 2010; Mundt 2011). Peer social networks’ effects suggest that peer education could be an effective primary prevention strategy.

Peer education strategies employ various theories including social learning, diffusion of innovations, and social role and expectations (Strange 2006). The choice of peer educators may depend to some extent on the theoretical bases of the preventive health intervention (Strange 2006). However, it is not clear who makes the “best” peer educator. Demographic similarity of the educator to the target population is important; however, knowledge of the topic, effective communication skills, and personality may be more important (Strange 2006).

Adolescents with more frequent church attendance represent a potential pool of peer educators who may be predisposed and motivated to offer anti-drinking and risk reduction messages to other students. Rather than the non-drinking students, those frequent church attending students who have some alcohol consumption experience might be more knowledgeable peer educators. A possible limitation to the use of adolescent church attenders as peer educators is that it is uncertain to what extent the “church” and “secular” adolescent social networks overlap. Church-attending adolescents with some drinking experiences might be “bridges” between different social networks and strategically useful in stimulating a wider diffusion of health promotion messages (Valente 2012).

Jernigan observes that alcohol-related social and religious movements have been “among the most powerful catalysts in developing societies … in reducing the rate of alcohol-related problems” (2002, p. 23). However, public health alliances with these movements have been problematic (Jernigan 2002). As recently observed about faith-based HIV programs, the presence of judgmental attitudes in religious programs can impede their successful integration into public health programs (Derose et al. 2010). Disagreements and tensions over institutional values between religious and health leaders limit their ability to work collaboratively (Derose et al. 2010). Given the inverse association between church attendance and alcohol consumption, another possible explanation for a loss of program efficacy could be that the social networks associated with the religious environment are more receptive to the anti-alcohol messages than the general public.

The levels of adolescent alcohol consumption in this study were comparable to other published reports. The levels were similar or less than in other low- and middle-income countries (Assanangkornchai et al. 2009; Piko and Fitzpatrick 2004; Reda et al. 2012; Sanchez et al. 2011; Zarzar et al. 2012) and similar to developed countries (Mäkelä et al. 2006; Melchior et al. 2008), though direct comparison is difficult because of how the data are reported and methodological differences (such as variant definitions of binge drinking and standard drinks) (Devos-Comby and Lange 2008).

A recent study of Dominican eighth, tenth, and twelfth graders found that 68.3 % initiated alcohol use before completing 15 years of age (similar to the 70.3 % estimate in this report) and found the identical 31.4 % rate of current drinkers (Cáceres Ureña et al. 2009).

The proportions of current drinkers in the present study were equal for the young men and women, even though the women had fewer episodes of inebriation and less average consumption than the male students. Other studies in Latin America find that men drink more regularly and in greater quantity than women (Cáceres Ureña et al. 2009; Kerr-Corrêa et al. 2005; Romero Mendoza et al. 2005; Wilsnack et al. 2005), though this may be changing as gender roles and opportunities change in Latin America for younger women in particular (Kerr-Corrêa et al. 2005; Romero Mendoza et al. 2005). Young women in the Dominican Republic are more likely to concentrate their drinking on weekends (Cáceres Ureña et al. 2009), a pattern that can be associated with heavy episodic drinking (Jernigan 2002). Young women in this study were not different than the young men in their self-reported episodic heavy drinking, a pattern also observed in younger adults in Europe (Mäkelä et al. 2006). Young women may have a risk equal to that of their male counterparts for complications of heavy episodic drinking such as accidents and unintentional injuries, a principal health risk for young men that drink (Ye and Cherpitel 2009).

Age limits on drinking are wide-spread, but less widely enforced (Jernigan 2002; Monteiro 2007). About three-fourths of the students indicating that they bought their alcohol themselves were underage in this study; more than 80 % of current drinkers in this study were underage. Estimates are that underage drinkers comprise about 10 % of the global alcohol market (Romero Mendoza et al. 2005). Enforcement of the minimal age for alcohol purchase might decrease underage drinking (Johnson et al. 1988; Monteiro 2007), though local conditions of limited resources and higher law enforcement priorities may hamper those efforts (Jernigan 2002). In addition, increased exposure of juveniles to alcohol occurs in tourist areas (Guilamo-Ramos et al. 2011). Analysis of alcohol promotion shows that young drinkers and women are a focus of alcohol advertising (Yoast et al. 2002) and that advertising and media are effective in increasing adolescent alcohol use (Anderson et al. 2009; Smith and Foxcraft 2009).

There are possible sources of bias in this study (Browne 2005a, b; Smith et al. 2005b). The sample population includes only those students attending school. Mandatory education in the Dominican Republic stops after the eighth grade. Accordingly, those young people not electing to continue in school are not represented in this study. The informed consent process identified the questionnaire’s source as a church-affiliated clinic. Some students may have tried to answer the questions the way they thought was expected and produced a “courtesy bias.” To the extent that some students conceive of alcohol use as bad, they might have “faked good” and under-reported their alcohol use; others may have “faked bad” and over-reported their alcohol use. Recall bias could have occurred, though young respondents are reasonably accurate in their self-reported drinking (Northcote and Livingston 2011). There were relatively few non-responders, but a bias arising from those who chose not to answer-specific items cannot be excluded.

Two secular private schools declined participation. One of these schools had the highest tuition of any of the non-public schools, being more than twice the value at the second most expensive school. The other school that declined participation had the third highest tuition costs. The decision by these two schools not to participate may have produced a socio-economic bias in the data. Higher material affluence is associated with more experimental alcohol use among adolescents in Ghana (Doku et al. 2012).

## Conclusions

The high proportion of under-age current drinkers in this study supported the use of regulatory policy approaches. The equal representation of male and female students among current drinkers and heavy episodic drinkers suggested that changing social norms may be placing young women at increasing risk of alcohol-related problems. This study found a relationship between the frequency of students’ church attendance and levels of alcohol consumption and associated risks.

The challenge is in translating the various observations from this and other studies into effective primary prevention programs to decrease alcohol risks for youth. This study suggests that a better understanding of the interactions between drinking and non-drinking adolescent peer networks—as well as the determinants of individual status and popularity within those networks—will be useful. Church-attending youth may represent a source of peers willing and motivated to participate in primary prevention activities. Additional qualitative studies (such as focus groups, analyzing adolescent narratives, and anthropological thick descriptions) could enhance the understanding of the important factors and their meanings within adolescent social networks.

## Appendix: Encuesta sobre el uso de alcohol

[Institucional identifying information removed]

El propósito de esta encuesta es describir el uso de alcohol en los estudiantes del séptimo curso hasta el cuarto de bachillerato. Hasta ahora, no hay descripciones de las costumbres de los jóvenes en la República Dominicana relacionado con el alcohol. Está encuesta es una investigación para explorar los comportamientos y actitudes de los jóvenes Dominicanos. Tu participación y tus respuestas serán importantes para entender y evaluar la situación actual.

Tus respuestas son anónimas y confidenciales. Entonces, ***no escriba tu nombre*** en esta encuesta. Resúmenes de los resultados de las encuestas no van a identificar a los individuos. Las respuestas que suministres serán mantenidas en secreto. Nadie conocerá tus respuestas.

Asegúrate de leer cada pregunta. Contesta a las preguntas basado en lo que realmente sabes o haces. No hay respuestas correctas ni incorrectas.

La realización de la encuesta es voluntaria. Tus notas o calificaciones no se afectarán si contestas o no a las preguntas. Si no quieres responder a una pregunta, simplemente déjala en blanco.

Por favor, indica tus respuestas en cada hoja con círculos o una marca clara según prefieres.

La encuesta demanda menos de 20 minutos para llenarla. Cuando termines con la encuesta, favor ponerla en el sobre y devolverla.

1. Tu edad: _____________ años
2. Tu género:
  1. Masculino
  2. Femenino
3. Tu curso:
  1. Séptimo (7^mo^)
  2. Octavo (8^vo^)
  3. Primero de bachillerato: (1^ro^ bachillerato)
  4. Segundo de bachillerato: (2^do^ bachillerato)
  5. Tercero de bachillerato: (3^ro^ bachillerato)
  6. Cuarto de bachillerato: (4^to^ bachillerato)
4. Asistes tu a una iglesia:
  1. Nunca
  2. A veces
  3. Cada mes más o menos
  4. Cada semana más o menos
  5. Más de una vez por semana
5. Algunos miembros de tu familia asisten a una iglesia:
  1. Nunca
  2. A veces
  3. Cada mes más o menos
  4. Cada semana más o menos
  5. Más de una vez por semana

**Las próximas 6 preguntas se refieren al consumo de bebidas alcohólicas. Esto incluye la ingestión de cerveza y ron, entre otros.**

**El consumo de alcohol** ***no incluye*** **beber** ***unos pocos sorbos*** **de vino en actividades religiosas.**

**Una “bebida entera” es un vaso de vino (11/3 onzas, 40 ml), una botella o lata de cerveza (10 onzas, 330 ml como una Presidente pequeña), o un vaso destilado (11/3 onzas, 40 ml)**

6. ¿Qué edad tenías cuando tomaste tu primera bebida de alcohol, algo más que unos pocos sorbos?
  1. Nunca he bebido alcohol aparte de unos pocos sorbos
  2. 7 años o menos
  3. 8 ó 9 años
  4. 10 ó 11 años
  5. 12 ó 13 años
  6. 14 ó 15 años
  7. 16 años ó más
7. ¿Qué tiempo hace que tomaste tu última bebida alcohólica?
  1. No tome alcohol
  2. Menos de una semana
  3. De una semana hasta un mes
  4. De un mes hasta dos meses
  5. De dos meses hasta tres meses
  6. De tres meses o más tiempo
8. **Durante los últimos 30 días**, ¿en cuántos días tomaste al menos una bebida entera que contenía alcohol?
  1. 0 días (No tomé alcohol en los últimos 30 días)
  2. 1 ó 2 días
  3. 3 a 5 días
  4. 6 a 9 días
  5. 10 a 19 días
  6. 20 a 29 días
  7. Los 30 días
9. **Durante los últimos 30 días**, ***en los días en que tomaste alcohol***, ¿cuántos bebidas alcohólicas enteras tomaste normalmente **por día**?
  1. No tomé alcohol durante los últimos 30 días
  2. Menos de una bebida entera
  3. 1 bebida entera por día
  4. 2 bebidas enteras por día
  5. 3 bebidas enteras por día
  6. 4 bebidas enteras por día
  7. 5 bebidas enteras ó más por día
10. Si eres una chica, vaya a la pregunta **para** **hembras**.
  Si eres un chico, vaya a la pregunta **para** **varones**.

**para** **hembras**:

- **Durante los últimos 30 días,** ¿cuántas veces has tomado cuatro (4) o más bebidas alcohólicas en un sólo día?
  1. Nunca
  2. Una vez (1 vez)
  3. De dos a cuatro veces (de 2 a 4 veces)
  4. Más de cuatro veces (más de 4 veces)

**para** **varones**:

- **Durante los últimos 30 días,** ¿cuántas veces has tomado cinco (5) o más bebidas alcohólicas en un sólo día?
  1. Nunca
  2. Una vez (1 vez)
  3. De dos a cuatro veces (de 2 a 4 veces)
  4. Más de cuatro veces (más de 4 veces)

11. **Durante los últimos 30 días**, ¿cómo conseguiste normalmente el alcohol que tomaste?
  **SELECCIONA SÓLO UNA RESPUESTA**
  1. No tomé alcohol durante los últimos 30 días
  2. Lo compré en una tienda, un mercado o en la calle
  3. Le di dinero a otra persona para que lo comprara por mí
  4. Lo conseguí de mis amigos
  5. Lo conseguí en mi casa
  6. Lo robé
  7. Lo hice yo mismo
  8. Lo conseguí de otra manera

**Tambalearse cuando uno camina, no ser capaz de hablar correctamente y vomitar son algunos signos de tener una borrachera.**

12. Durante tu vida, ¿cuántas veces tomaste tanto alcohol que llegaste a emborracharte?
  1. Ninguna – 0 veces
  2. 1 ó 2 veces
  3. 3 a 9 veces
  4. 10 ó más veces
13. Durante tu vida, ¿cuántas veces has tenido problemas con tu familia o amigos, has faltado a la escuela o te has metido en peleas como resultado de tomar alcohol?
  1. Ninguna – 0 veces
  2. 1 ó 2 veces
  3. 3 a 9 veces
  4. 10 ó más veces
14. En tu casa, ¿normalmente se almacenan cervezas, ron, u otras bebidas alcohólicas?
  1. Sí, siempre o casi siempre hay bebidas alcohólicas en la casa
  2. A veces hay bebidas alcohólicas en la casa
  3. No, nunca hay bebidas alcohólicas en la casa
15. ¿Cuántos miembros de tu hogar toman bebidas alcohólicas?
  1. Nadie
  2. Algunos (de 1 a 3 personas)
  3. Más de 3 personas
16. ¿Con cuál frecuencia toman bebidas alcohólicas algunos miembros de tu hogar?
  1. Nunca
  2. Menos de una vez por mes
  3. Algunos veces por mes
  4. 1-2 días por semana o sólo en los fines de las semanas
  5. 3-5 días por semana
  6. Todos los días de la semana
